# Prescribing trends of oral anticoagulants in England over the last decade: a focus on new and old drugs and adverse events reporting

**DOI:** 10.1007/s11239-021-02416-4

**Published:** 2021-03-05

**Authors:** Saima Afzal, Syed Tabish Razi Zaidi, Hamid A. Merchant, Zaheer-Ud-Din Babar, Syed Shahzad Hasan

**Affiliations:** 1 Department of Pharmacy, School of Applied Sciences, University of Huddersfield, Huddersfield, UK; 2 School of Healthcare, University of Leeds, Leeds, UK; 3 Leeds Teaching Hospitals NHS Trust, Leeds, UK

**Keywords:** Direct-acting oral anticoagulants (DOACs), Prescriptions trends, Warfarin, INR, Atrial fibrillation

## Abstract

Direct-acting oral anticoagulants (DOACs) are replacing conventional VKA (vitamin K antagonist, i.e., warfarin) for various indications where a therapeutic anticoagulant effect is desired. We evaluated the prescribing patterns of the DOACs and warfarin, cost implications of the increasing DOACs prescribing, and deduce the reporting of serious and fatal events, during 2009–2019 in primary care England. Prescriptions and fatal or serious adverse events reporting data, between 2009 and 2019 were analysed, using linear regression to examine the trends in prescriptions, costs, and serious and fatal events reporting. We also compared the prescribing trends of four direct-acting oral anticoagulants and warfarin, normalised to per 1000 clinical commissioning group (CCG) patient population for the year 2019 to better understand the regional differences in DOACs prescribing. The overall use of any DOACs (as a proportion of total anticoagulants) increased from 16% in 2015 to 62% in 2019 with an average increase of 87% (95% CI 83.1, 90.5) per year. The reporting of serious and fatal events associated with DOACs decreased by 6% (95% CI 12.5, − 0.1) per year. Apixaban is by far the most prescribed with an average drug cost increasing to 156% (95% CI 140, 172) per year. In England, the lowest anticoagulant prescribing region was Greater London whereas the highest prescribing regions were Yorkshire and Humber for DOACs and the East Midlands for warfarin. Interestingly, Lancashire, Merseyside, and Cheshire showed a higher usage for warfarin over DOACs. The differing prescription patterns could be a result of changes in national guidelines and increasing population. Nevertheless, DOACs appear to make an increasing contribution to total anticoagulant prescription items and costs.

**Supplementary Information:**

The online version of this article (10.1007/s11239-021-02416-4) contains supplementary material, which is available to authorized users.

## Highlights

- DOACs are becoming increasingly popular than the VKA (warfarin) for a range of conditions.
- We analysed trends in prescriptions, costs, and ADR reporting of DOACs and VKA during 2009–2019.
- The DOACs account for 62% of all oral anticoagulant prescriptions in 2019 compared with 16% in 2015. Warfarin use was declined since 2015 and apixaban is the most used DOAC, and closing the warfarin gap.
- Except for edoxaban and apixaban, the reporting of serious and fatal events associated with DOACs decreased by 6% per year. The higher proportion of ADRs attributed to DOACs is, however, associated with the higher usage of DOACs in recent years than the warfarin. 
- Interestingly, the number of serious or fatal ADRs per million items for warfarin are doubled in 2019 than what it was about 10 years ago.

## Introduction

Direct-acting Oral Anticoagulants (DOAC) (apixaban, rivaroxaban, dabigatran, and edoxaban) are becoming increasingly popular in the prevention and treatment of a range of conditions such as stroke prevention in atrial fibrillation, venous thromboembolism, and deep vein thrombosis [1].

Dabigatran; the first DOAC was approved in the UK in 2008 and was shortly followed by the approval of rivaroxaban in the same year [2], apixaban in 2011 [3], and edoxaban in 2015 [4, 5]. Despite warfarin being used for almost 70 years [6], DOACs are slowly but surely replacing warfarin [7]. DOACs are favoured over vitamin K antagonists (warfarin) as they require less frequent monitoring, which deems them more convenient and cost-efficient [8, 9]. Furthermore, they have pharmacological advantages over warfarin as they have a faster onset of action, predictable pharmacokinetics, and fewer drug and food interactions [9]. The recent findings from the PROSPER study show that older patients with atrial fibrillation and ischemic stroke receiving DOACs were less likely to experience major adverse cardiovascular events and had fewer deaths and readmissions compared with warfarin [8].

Currently, there are at least two studies published in the UK on the comparison of prescribing patterns for warfarin and the direct oral anticoagulants. Connelly published a comparison of total prescriptions dispensed for the first three approved DOACs compared to warfarin [10]. However, the study only compared the data up until 2015, this was before the EU approval of edoxaban. The study concluded that the total usage of warfarin in 2015 was 11.6 million prescriptions, compared to 1.5 million in total for rivaroxaban; 0.7 million for apixaban, and 0.4 million for dabigatran. A recent study by Ho et al. also noticed a significant increase in the prescriptions of DOACs from 9% in 2014 to 74% in 2019; the magnificent increase was consistent across almost all of the evaluated clinical commissioning groups (CCGs) [11].

As mentioned, the use of warfarin has been superseded by the newly developed DOACs, particularly in the treatment of venous thromboembolism and the prevention of stroke in patients with non-valvular atrial fibrillation [12]. There was one study by Loo et al., that reported DOACs utilisation trends using patient-level data between 2009 and 2015, but the authors did not present data on edoxaban, adverse drug reaction (ADR) reporting, and the cost implications [5]. No population-level quantitative analysis is available comparing DOACs and warfarin with regards to their ADR reports and associated drug costs analysis. Therefore, this study will aid in providing an understanding of the potential impact DOACs have had on the prescribing rates and the impact this has had with regards to the cost, as well as the ADR profile of these drugs.

## Methods

This study evaluates prescribing trends, costs, and serious and fatal events reported for four direct-acting oral anticoagulants (DOACs) and warfarin in primary care England, to understand any consequent changes in use and NHS prescription reimbursements. Prescribing data for five oral anticoagulants between January 2009 and December 2019 was obtained from the Prescription Cost Analysis [13]. This data highlights the quantities of each unit of drug and prescription items dispensed, thus allowing for the analysis of prescription trends. Table 1 expresses all drugs, strengths, and dosage forms used in this study.

**Table 1:** Presentations analysed

Drug name	Brand	Strength	Dosage forms
Warfarin	Warfarin sodium, marevan	0.5 mg, 1 mg, 3 mg, 5 mg,	Tablet, suspension, liquid
Dabigatran	Pradaxa ®	75 mg, 110 mg,150 mg	Capsule
Rivaroxaban	Xarelto ®	2.5 mg, 10 mg, 15 mg, 20 mg	Tablet
Apixaban	Eliquis ®	2.5 mg, 5 mg	Tablet
Edoxaban	Lixiana ®	15 mg, 30 mg, 60 mg	Tablet

The NHS BSA (Business Services Authority) is a corporation involving what were originally five separate organisations—one of which was the Prescription Pricing Authority [13]. The BSA documents numerous types of data and information regarding medicines, though also conducts the function of ensuring money in the NHS is well spent—advising numerous healthcare professionals and companies on where money may be spent more beneficially. The data is arranged and separated by month, including every single prescription item—separating each drug into an individual formulation, and strength. The dispensing data are also arranged according to the type of dispenser—pharmacies, appliance contractors, dispensing doctors, and medicines sold under private administration—though all data were accumulated for use in this study. This database does not collate private prescriptions dispensed in a community or a hospital [13].

The number of prescription items and net ingredient costs of all prescriptions dispensed in the community in England is specified in the Prescription Cost Analysis database [13]. The drugs dispensed are listed by the British National Formulary (BNF) therapeutic class using the classification system in-use prior to the BNF edition 70. The PCA data contains a multitude of data, such as standard quantity units, items dispensed, costs, etc. The number of total units dispensed (e.g. tablets, capsules, millilitres)—specific for each strength and brand and net ingredient cost—the price of the medicines as outlined by the drug tariff, or manufacturer or wholesaler (where appropriate) were recorded. The net ingredient cost is the price before any discounts are applied and does not include any dispensing costs or fees. It also does not include any adjustment for income obtained where a prescription charge is paid at the time the prescription is dispensed or where the patient has purchased a pre-payment certificate. The data is internally audited to 99% accuracy that is at least 99 percent of prescriptions are recorded accurately and are available online [13–15].

The serious and fatal ADR reports associated with DOACs and warfarin were obtained from the Medicines and Healthcare Products Regulatory Agency (MHRA) Yellow Card Scheme, reported between January 2009 and December 2019 [16]. The MHRA collects data for adverse drug events via the Yellow card scheme in the UK. The yellow card scheme is a nationally recognised recording database where all patients and Healthcare professionals able to report any adverse effects experienced with their medicines. The Scheme also allows for the reporting of fake medicines, as well as ADR’s experience with device use. A suspected ADR report is considered ‘serious’ according to the reporter who considers the reaction to be serious or the reaction term itself is considered serious in MHRA medical dictionary [16].

The extracted data were analysed using Microsoft Excel and SPSS. The monthly data obtained from the PCA was extracted and tabulated according to each DOAC and Warfarin, and the total quantities for each drug per month were calculated—summarising all formulations and strengths [13]. The quantities for each month were then summed to find the total quantities per year in units of thousands.

The trends in both prescriptions and costs of different categories of drugs over 10-year (2009–2019) and 5-year (2015–2019) periods were examined. The data for the first approved DOAC (dabigatran) and last approved DOAC (edoxaban) were available from 2008 and 2015, respectively. For analysis and presentation of costs, we adjusted for inflation in years before 2019, using the inflation calculator from the Bank of England website [17]. We computed the proportion of total prescription numbers and costs accounted for by all oral anticoagulant medication combined in both 2015 and 2019, and we examined the contribution made by different categories of anticoagulants to prescriptions and costs in both years. We used linear regression analysis with the year as the independent variable and prescription items (quantity) and costs as the dependent variables, using data from each year (from 2015 to 2019 when all four DOACs were available). We calculated the average annual percentage increase by dividing the regression coefficient by the baseline prescriptions or costs from 2015 [18]. To establish a regional comparison, a geographical map was plotted using Microsoft Excel, taking the total prescription items dispensed per 1000 CCG patient population for the point of latitude [19].

## Results

**Fig. 1:**
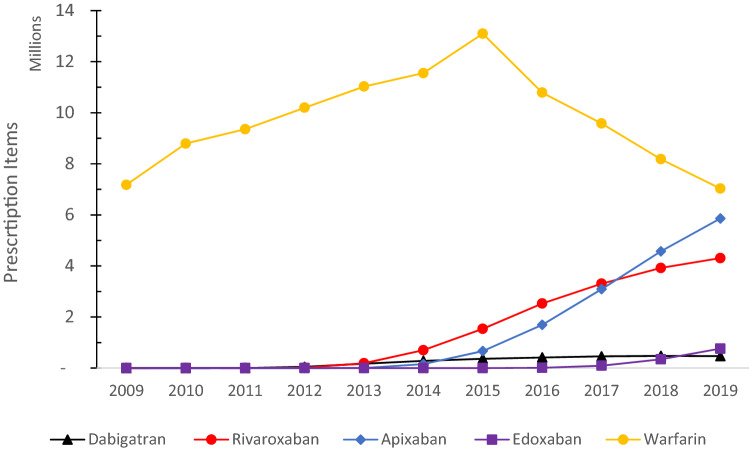
Trends of DOACs and Warfarin use (in millions) in the UK over 11 years (2009–2019)

Figure 1 shows trends in prescriptions for the prescribed anticoagulants between January 2009 and December 2019. Warfarin consumption was reduced considerably between 2009 and 2019. There was a sharp decline in warfarin use after 2015. In 2009, only two DOACs, dabigatran, and rivaroxaban were available, and their use was negligible compared to warfarin (0.01% vs. 99.99%). The oral anticoagulants made up 1.8% of all prescription items in 2019 compared with 1.4% in 2015 (Table 2). The direct oral anticoagulants (DOACs) accounted for 61.8% of all oral anticoagulants prescription items in 2019 compared with 16.4% of all prescription items in 2015. Apixaban is by far the most prescribed DOAC of the group and is closing the gap with warfarin prescriptions (32% in 2015 vs. 38% in 2019). Specifically, the use of apixaban has increased from 4.3% in 2015 to 31.8% in 2019.

**Table 2:** Prescription items dispensed and costs of anticoagulant drugs, 2015 and 2019

Drug class, n (%)	Thousands of prescription items		Inflation-adjusted costs, £000 s	Costs, £000 s
	2015	2019	2015	2019
Drugs, *n* (%)^a^				
Apixaban	667.2 (4.3)	5859.7 (31.8)	41,626.9 (21.8)	268,848.2 (50.0)
Dabigatran	366.0 (2.3)	467.6 (2.5)	25,657.7 (13.5)	22,241.1 (4.1)
Rivaroxiban	1537.7 (9.8)	4307.3 (23.4)	93,810.2 (49.3)	201,868.9 (37.5)
Edoxaban	0.2 (< 1)	763.7 (4.1)	16.7 (< 1)	34,490.1 (6.4)
Warfarin	13,098.1 (83.6)	7030.3 (38.1)	29,361.5 (15.4)	10,148.5 (1.9)
Total anticoagulant drugs, n	15,669.3	18,428.6	190,473.1	537,596.8
Total DOACs, n (%)	2571.2 (16.4)	11,398.30 (61.8)	161,111.5 (84.6)	527,448.3 (98.1)
Total BNF listed drugs excluding anticoagulants, n	1,068,006.3	1,006,638.0	9,874,756.4	6,979,0003.7
Total BNF listed drugs	1,083,675.6	1,025,066.6	10,060,328.7	8,390,797.5
Anticoagulant drugs as proportion of BNF total, %	1.4	1.8	1.8	6.4

^a^Percentage is the % of all anticoagulant prescription items

Table 3 presents the results of regression analysis of yearly trends in prescriptions dispensed and associated costs. Overall, prescription items for oral anticoagulant drugs increased by about 4.8% (95% CI 2.1–7.6) per year on average between 2015 and 2019. The prescription items for DOACs increased by about 86.8% (95% CI 83.1–90.5). The increasing trends for all DOACs were statistically significant.

**Table 3:** Regression analysis of yearly trends in prescriptions and costs, 2015–2019

Drugs (baseline year)	Prescription trends		Prescriptions, mean change per year as % of baseline^a^ (95% CI)	Cost trends		Costs, mean change per year as % of baseline^a^ (95% CI)
	Regression coefficient (95% CI)	*p*		Regression coefficient (95% CI)	*p*	
Apixaban (2015)	1326.42 (1,186.81, 1,466.04)	0.001	198.80 (177.88, 219.73)	58,070.08 (52,179.74, 63,960.42)	0.001	155.82 (140.01, 171.62)
Dabigatran (2015)	26.63 (4.08, 49.49)	0.033	7.28 (1.12, 13.52)	− 24.77 (− 989.82, 940.28)	0.940	− 0.11 (− 4.31, 4.09)
Edoxaban (2015)	185.87 (32.25, 339.50)	0.031	92,935.00 (16,125.00, 169,750.00)	8,281.80 (1253.69, 15,309.91)	0.033	57,115.86 (8528.50, 104,149.05)
Rivaroxaban (2015)	693.20 (479.85, 906.56)	0.002	45.08 (31.21, 58.96)	29,175.42 (20,388.79, 37,962.05)	0.002	34.74 (24.28, 45.20)
Warfarin (2015)	− 1474.32 (− 1,858.85, − 1,089.78)	0.001	− 11.26 (− 14.19, − 8.32)	− 4157.31 (− 7448.61, − 866.02)	0.033	− 15.82 (− 28.34, − 3.29)
Total anticoagulants	757.80 (322.29, 1,193.31)	0.012	4.84 (2.06, 7.62)	91,345.22 (78,940.43, 103,750.08)	0.001	53.57 (46.29, 60.84)
Total DOACs	2,232.11 (2,137.53, 2,326.69)	0.001	86.81 (83.13, 90.49)	95,502.52 (85,410.65, 105,594.40)	0.001	66.21 (59.21, 73.21)

^a^ = % change was calculated by dividing the regression coefficient by baseline prescriptions or costs from 2015 as given in Table 1. 2015 was used because all 5 drugs were available from 2015

By 2019, oral anticoagulants accounted for 6.7% of all prescription drug costs compared with 1.8% in 2015. The direct oral anticoagulants (DOACs) accounted for 61.8% of all oral anticoagulant prescription items and 98% of all (oral anticoagulants) costs in 2019. In terms of costs, apixaban overtook rivaroxaban as the most expensive drug by 2019, accounting for 50% of the costs of oral anticoagulants in that year.

**Fig. 2:**
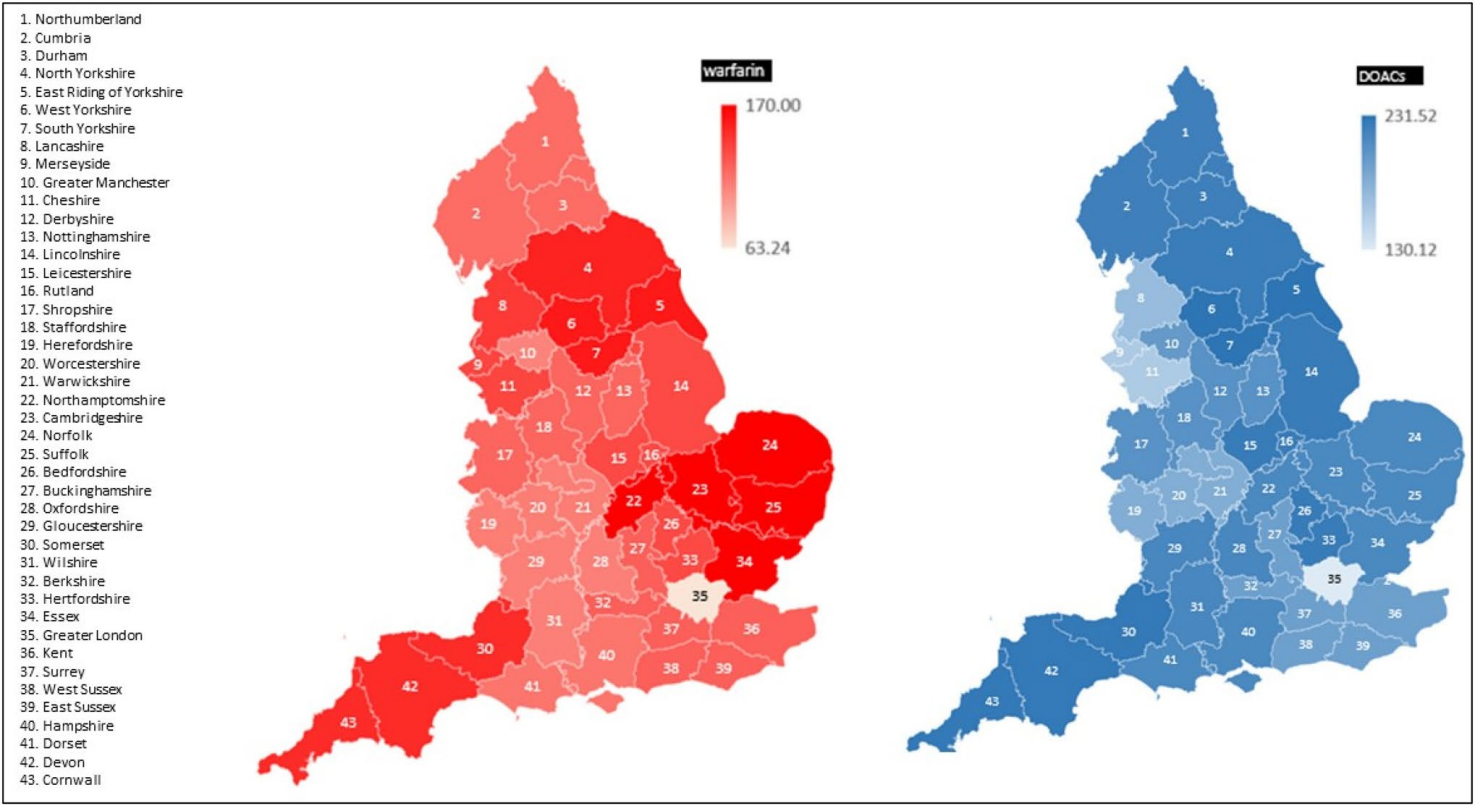
Total prescription items for warfarin and DOACS, normalised to per 1000 CCG population for the year 2019 (Red = warfarin; Blue = DOACs)

The geographical differences were also evident (Fig. 2) and highlighted the regions with the highest and lowest rates of anticoagulant prescriptions shown by the shades of red (warfarin) and blue (DOACS). The lowest anticoagulant prescribing region was Greater London (warfarin = 63.24 per 1000 vs. DOACs = 130.12 per 1000 CCG population). The highest prescribing regions were Yorkshire & Humber for DOACs (231.52 per 1000) and the East Midlands for warfarin (170.0 per 1,000). The highest prescribing CCGs were in Leeds (range: 28.74–33.29 per 1000) for warfarin and Gloucestershire for DOACs (range 51.88–63.37 per 1000).

**Fig. 3:**
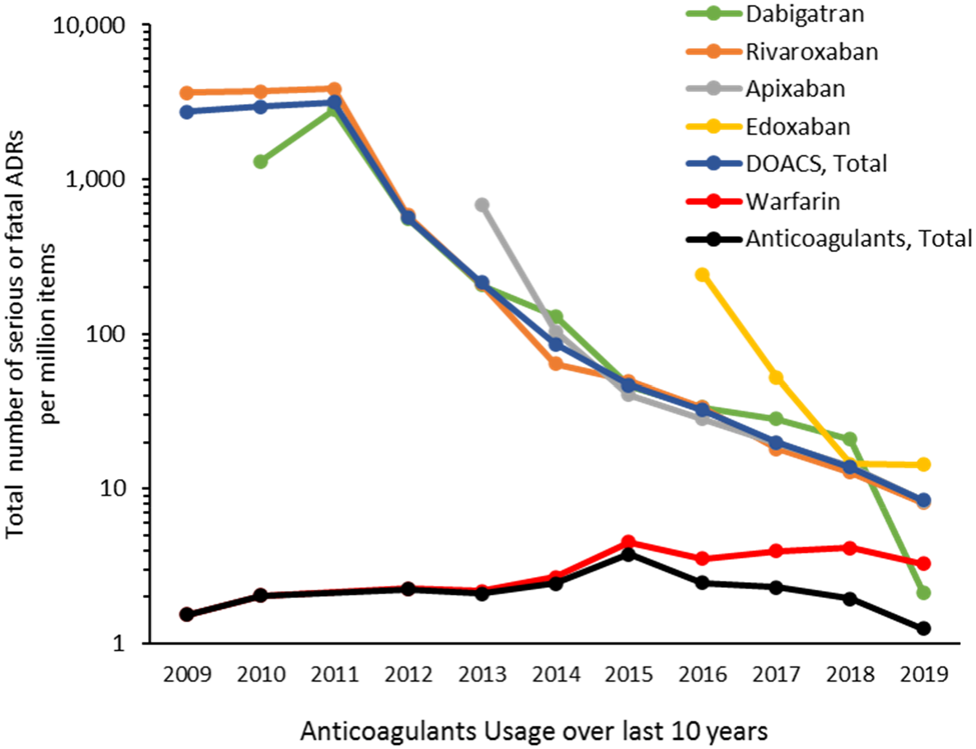
Serious/fatal adverse drug reactions (ADRs) for various anticoagulant drugs normalised to the number of anticoagulant items prescribed during the last 11 years

Figures 3 and S2 display the trend of the total number of ADRs and the total number of serious ADRs with anticoagulant usage over the last 11 years, normalized to the respective number of prescription items. As of 2019, the drug with the highest number of ADR reports (serious and fatal) in England was apixaban (643 events reported), ahead of rivaroxaban. However, this may be because apixaban was prescribed more than rivaroxaban; the number of ADRs per 100,000 items for both drugs are not dissimilar. As of 2019, dabigatran has the least number of ADR reports and had a steady decline in the number of ADR reports from 2013 onwards, from 338 serious or fatal events reported in 2013 to 52 events reported in 2019. This may also be linked to drug usage, and the number of ADRs normalised to the number of prescription items for dabigatran is similar to rivaroxaban and apixaban. Edoxaban, however, has continuously increased the number of ADR reports and now stands with warfarin in the number of ADR reports (201 events vs. 215 events, in 2019), but has demonstrated eight times the number of ADRs per item prescribed as compared to the number of ADRs with warfarin. DOACs made up 81% of all fatal events reported for oral anticoagulant drugs in 2019 compared with 19% reported in 2009. By 2019, warfarin accounted for 19% of all fatal events reported for oral anticoagulant drugs. The higher proportion of ADRs attributed to DOACs is, however, associated with the higher usage of DOACs in recent years than the warfarin.

There was a statistically significant downward trend of serious or fatal ADRs associated with DOACs and a simultaneous increase in serious or fatal ADRs with warfarin. Overall, there is a declining trend of serious ADRs with total anticoagulant drugs. The overall reporting of serious and fatal events decreased for all anticoagulant drugs by 4% (95% CI − 9.79–2.15, p = 0.115) per year on average between 2015 and 2019 (Figure S1). Whereas, the reporting of serious and fatal events associated with DOACs decreased by 6% (95% CI 12.5–0.11). Interestingly, the number of serious or fatal ADRs per million items for warfarin are doubled in 2019 than what it was about 10 years ago.

## Discussion

The present study indicates a steady increase in overall anticoagulant prescriptions over the last five years. Warfarin use reached a peak in 2015 and since then gradually declined on the establishment of dabigatran, apixaban, rivaroxaban, and edoxaban as clinically comparable treatment options. By 2019, DOAC prescriptions accounted for more than half of all anticoagulant prescriptions. Apixaban is by far the most prescribed DOAC of the group (despite dabigatran being the oldest DOAC on the market) and is closing the gap with warfarin prescriptions. Malik et al. evaluated the safety and efficacy of DOACs and warfarin in older patients with non-valvular atrial fibrillation and reported an improved efficacy compared to warfarin in reducing stroke or systemic embolization [20]. All DOACs were associated with reduced intracranial bleeding compared to warfarin.

The prescription items for DOACs were generally on the rise across different CCGs in England, albeit we noticed some geographical differences. The regional differences were thought to be linked with local policies and practices. Ho et al. examined the association between local policies and local prescribing practices [11]. The local policies were categorised into no recommendation, warfarin first-line, or identification of a preferred DOAC. They found a weak association between the types of policies and local prescribing practices; an association of local policies with the choice of individual DOAC within the category of DOACs was moderate [11]. The authors concluded that there was no influence of local recommendations on choices between DOACs and warfarin, but these recommendations still play an important role in determining the choice of specific DOAC in a region [11].

By the November 2020, prescription items dispensed for DOACs were more than double the number of warfarin items (12.1 million vs 5.3 million) costing extra £561 million. In the same period, apixaban has exceeded warfarin numbers (6.3 million vs 5.3 million prescription items) and became the most widely prescribed oral anticoagulant in the primary care England. The anticoagulant use during 2020 may have been influenced due to COVID-19 associated coagulopathy. It is noteworthy that there was an increase in the prescription for DOACs over the years and a steady anticoagulant use in the UK is also evident. These observations offer significant public health implications and may highlight an increase in anticoagulant awareness over the last decade. It could also be due to an increase in the ageing population or an increase in population with progressively ill-health due to increased incidence of thrombotic conditions. This can also be attributed to a recognition of DOACs use in catheter ablation that is still a well-established rhythmic control strategy for patients with drug-refractory and symptomatic arrhythmias but is associated with a high risk of thromboembolism perioperatively. A meta-analysis by Wu et al. found comparable efficacy and safety of DOACs to warfarin in 11,686 patients undergoing catheter ablation for atrial fibrillation, albeit the risk for minor bleeding was lower with DOACs than warfarin [21]. The DOAC associated reduced risk of bleeding during catheter ablation for atrial fibrillation was also confirmed by Ge et al. in a systematic review and meta-analysis involving 12,644 patients [22]. Among DOACs, dabigatran, the first available DOAC, was the least prescribed DOAC. This could be due to the fact that it is less suitable in patients with renal impairment and licensed for fewer indications [23].

The data show that the total and serious (or fatal) ADRS have both significantly reduced over the last decade which can be attributed to better safety profile of DOAC drugs. Apixaban is by far the most prescribed agent among the DOACs due to its efficacy and safety, and could also be owed to the preferable dosing regimen and number of licensed indications. Another possible reason is their NICE recommendations in comparison to other agents which would appeal to the prescribers. In 2019, apixaban use accounted for more than a third of all anticoagulant prescriptions (32%). This, in turn, has led to an exponential increase in the reporting of adverse drug reactions with the MHRA yellow card scheme [16]. Apixaban ADRs were 60–70% more prevalent compared to warfarin during 2016–2019. Also, we noticed a large value for edoxaban showing high reporting of ADR associated with edoxaban. This can be attributed to the increased usage of DOCAS with time. Besides, there could be several other reasons to explain this significant rise in the reporting of ADRs. Apixaban and edoxaban are comparatively new drugs and as such are highlighted in the British National Formulary with a black triangle. This indicated to healthcare professionals that any ADRs related to this must be reported via the yellow card scheme. On the other hand, warfarin has been used for decades therefore healthcare professionals feel a degree of comfort with prescribing, and ADRs have been well documented and known to the users and prescribers. The lack of understanding and knowledge about new DOACs such as apixaban and edoxaban (less prescribing experience) may have resulted in the discovery of ADRs which were not previously known.

The Yellow card scheme is based on voluntary ADR reporting. There is a possibility of under-reporting of ADRs and this could occur due to lack of recognition of ADRs [24], or failure to carry out a report due to other reasons, such as lack of time [25]. Also, there have been reports of variability in the quality of Yellow Card completion, which can lead to wrong interpretation of the ADR [26]. Assessment of causality between a drug and an ADR is challenging that could lead to under-reporting of ADR therefore may not correlate with the incidence of ADRs. Nevertheless, reports obtained by the MHRA are not instantly documented, as they are assessed according to clinical trials and relevant literature, to ensure the validity or find a cause for the reports [16]. These assessments are conducted by healthcare professionals, therefore, all reports documented by the Yellow Card Scheme are ensured to be legitimate [16].

The PCA database provides comprehensive population-level data on NHS primary care prescriptions and costs in England [13]. However, some limitations should be considered when interpreting the findings of this study such as no patient-level details were available. This analysis of prescription trends and utilisation of oral anticoagulants is limited to the national prescription data available in England. The increasing prescriptions do not necessarily reflect an increasing number of medicine users since chronic use and an increase in population size would also increase prescription numbers and costs [18]. Moreover, the  figures do not include prescription issued or items commenced in private or secondary care, nor do these figures provide indications of patient-related factors such as concordance and satisfaction. Still, the present analysis is the first to examine data on anticoagulant drugs since the introduction of edoxaban in 2015, and to use statistical approaches to explore and compare trends in prescriptions, costs, and ADRs over time.

## Conclusion

This study noticed a growing increase in overall anticoagulant utilisation within NHS England over the years. It was also found that the DOACs prescriptions are on the increase and overtook traditional warfarin-based treatment. Warfarin use reached a peak in 2015 and has since then gradually declined with a significant increase in the use of DOACs. Apixaban was the most prescribed DOAC of the group and is seems to close the gap with warfarin prescriptions possibly due to its efficacy and safety profile.

## Supplementary Information

Below is the link to the electronic supplementary material.

Electronic supplementary material 1 (DOCX 69 kb)
