# Supplementary material for: Prescribing trends of oral anticoagulants in England over the last decade: a focus on new and old drugs and adverse events reporting
Source: J Thromb Thrombolysis. 2021 Mar 5;52(2):646–53. doi: 10.1007/s11239-021-02416-4 (PMC7933373; doi:10.1007/s11239-021-02416-4)
Supplement: Supplementary file 1 — Electronic supplementary material 1 (DOCX 69 kb) [file 11239_2021_2416_MOESM1_ESM.docx]

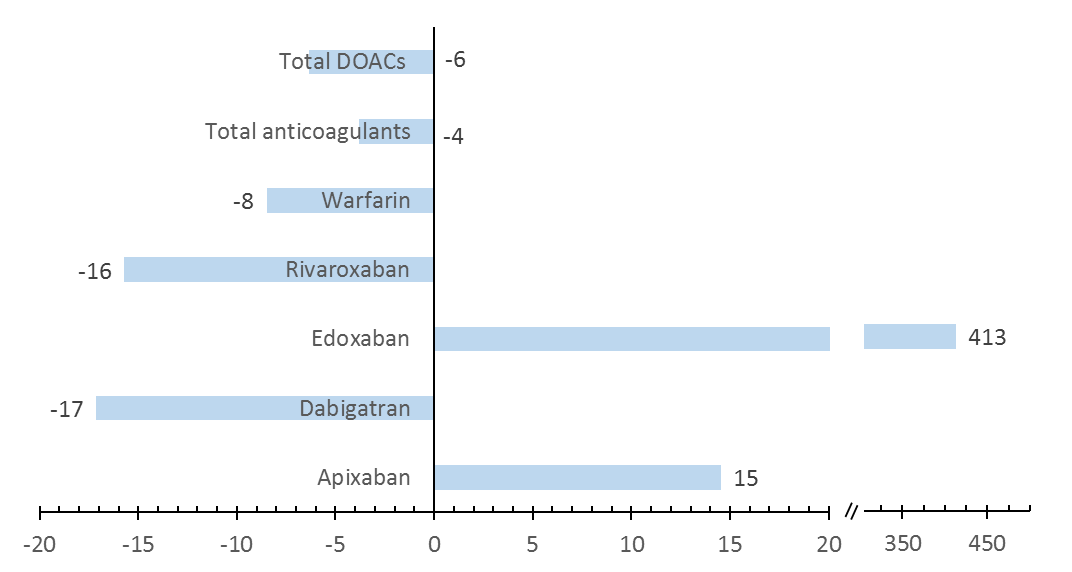


**FIGURE S1.** Average percentage change in ADR events per year (between 2015 – 2019) for anticoagulant drugs

The percent change was calculated by dividing the regression coefficient by baseline ADR from 2015.


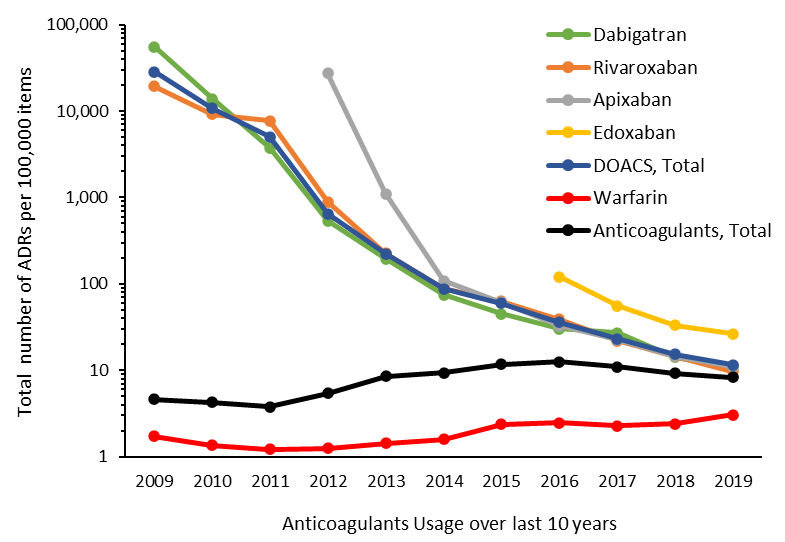


**FIGURE S2.** Total adverse drug reactions (ADRs) for various anticoagulant drugs normalised to the number of anticoagulant items prescribed during the last 10 years.
